# Left atrial strain reproducibility using vendor-dependent and vendor-independent software

**DOI:** 10.1186/s12947-019-0158-y

**Published:** 2019-05-15

**Authors:** Yu Wang, Zhilian Li, Hongwen Fei, Yongsen Yu, Siqi Ren, Qiongwen Lin, Hezhi Li, Yongwen Tang, Yuezheng Hou, Mingqi Li

**Affiliations:** 1 Department of Cardiology, Guangdong Cardiovascular Institute, Guangdong Academy of Medicine Sciences, Guangdong Provincial People’s Hospital, 106 Zhongshan Er Road, Guangzhou, 510100 China; 2 Shantou University Medical College, Shantou, Guangdong China; 3 Nansha Hospital, The first Hospital of Guangzhou, Guangzhou, China

## Abstract

**Background:**

Two-dimensional speckle-tracking echocardiography (2D-STE) enables objective assessment of left atrial (LA) deformation through the analysis of myocardial strain, which can be measured by different speckle-tracking software. The aim of this study was to compare the consistency of 3 different commercially available software, which include vendor-specific software for measuring left ventricle (VSS_LV_), vendor-independent software packages for measuring LV strain (VIS_LV_) and vendor-independent software packages for measuring LA strain (VIS_LA_).

**Methods:**

Sixty-four subjects (mean age: 44 ± 16 years, 50% males) underwent conventional echocardiograms using a GE Vivid 9 (GE Ultrasound, Horten, Norway) cardiac ultrasound system. Standard apical 4 and 2 chamber views of the left atrium were obtained in each subject with a frame-rate range of 40–71 frames/s. LA strain during the contraction phase (Sct), conduit phase (Scd), reservoir phase (Sr = Sct + Scd) were analyzed by 2 independent observers and 3 different software.

**Results:**

Sct, Scd, Sr were, respectively, − 11.26 ± 2.45%, − 16.77 ± 7.06%, and 28.03 ± 7.58% with VSS_LV_, − 14.77 ± 3.59%, − 23.17 ± 10.33%, and 38.23 ± 10.99% with VIS_LV_, and − 14.80 ± 3.88%, − 23.94 ± 10.48%, and 38.73 ± 11.56% when VIS_LA_ was used. A comparison of strain measurements between VSS_LV_ and VIS (VIS_LV_ and VIS_LA_) showed VIS had significantly smaller mean differences and narrower limits of agreement. Similar results were observed in the coefficient of variation (CV) for measurements between VSS_LV_ and VIS (VIS_LV_ and VIS_LA_). Comparison of the intra-class correlation coefficients (ICCs) indicated that measurement reliability was weaker with VSS_LV_ (ICC < 0.6) than with VIS (VIS_LV_ and VIS_LA_) (ICC > 0.9). For intra-observer ICCs, VIS_LA_ > VSS_LV_ = VIS_LV_. For inter-observer ICCs, VSS_LV_ > VIS_LA_ > VIS_LV_.

**Conclusions:**

Software measurement results of LA strain vary considerably. We recommended not measuring LA strain across vendor platforms.

**Electronic supplementary material:**

The online version of this article (10.1186/s12947-019-0158-y) contains supplementary material, which is available to authorized users.

## Introduction

LA is of hemodynamic importance for overall cardiac performance through reservoir, conduit, and booster pump functions [1–3]. The components of left atrial function are traditionally estimated using Doppler analysis of trans-mitral and pulmonary vein flow. However, the evaluation of LA function by Doppler analysis can be affected by left ventricle (LV) dysfunction, and is therefore limited [4–6]. Two-dimensional (2D) quantification of cardiac chamber size can be used to assess LA remodeling and function. M-mode echocardiography measured LA anteroposterior (AP) linear dimension only represents a single parameter of the left atrium LA [7, 8]. LA volume measured by 2D echocardiography reflects LA chamber size in all directions. However, the value heavily dependent on geometric assumptions [9]. Furthermore, the lack of a standardized methodology for three-dimensional (3D) echocardiography prevents the widespread use of 3D echocardiography to measure LA function [10, 11].

Because of the aforementioned drawbacks, there is increasing interest in speckle-tracking echocardiography (STE), which provides visualization of all phases of LA function [12, 13].

Initially, there was no specialized 2D-STE software for the assessment of LA deformation. Studies evaluating LA function used software designed for evaluation of the left ventricle (LV) [14]. It is controversial to measure LA strain without dedicated software [13, 15]. And there are a number of different commercially available software packages [16, 17]. Though consensus has been reached that the relative variation in LV strain measurement among different software should not exceed 10% before the technique can be recommended for clinical use [18], this has not been agreed upon for measurement of LA strain. As such, the widespread use of LA strain measurement could be hindered by the uncertainty of measuring strain with different software [19].

Thus, the purpose of this study was to compare the results of measuring LA strain with 3 different software packages.

## Methods

### Study population

Adult patients receiving echocardiography at the clinics of the Adult Echocardiography Lab of Guangdong Provincial People’s Hospital from December 2016 to September 2017 were recruited for the study. This study was approved by Research Ethics Committee of Guangdong Provincial People’s Hospital, Guangdong Academy of Medical Sciences. Inclusion criteria were: (1) older than 18 years; (2) sinus rhythm at examination; (3) agreed to participate in the study and provided written informed consent. Exclusion criteria were: (1) onset of atrial fibrillation during the examination; (2) valvular heart disease (moderate or severe heart valve stenosis or valve replacement); (3) implantation of a pacemaker or defibrillator; (4) poor image quality; (5) did not provide informed consent. Clinical data including a history of hypertension, diabetes mellitus, dyslipidemia, and smoking were collected trained research staff at the time of the first hospital admission.

We identified 99 participants. Of these, 35 subjects were excluded from analysis for not providing consent (*n* = 5), sinus rhythm turning into atrial fibrillation (*n* = 7) or inadequate imaging quality due to acquisition with unclear LA endocardium (*n* = 12), LA foreshortening (*n* = 11). The final study population consisted of 64 individuals (male/female, 32/32; mean age = 44.1 ± 16.1 years).

### Echocardiographic acquisition

Echocardiographic studies were performed using a GE Vivid 9 (GE Vingmed Ultrasound, Horten, Norway) echocardiograph system. Examinations were performed with subjects in the left lateral recumbent position. Apical 4- and 2-chamber views were obtained using conventional 2D gray scale echocardiography with an M5S probe (2 ~ 4 MHz), using a frame-rate of 40–71 frames/s, in accordance with current American Society of Echocardiography recommendations [20]. Both apical views used should be optimized in terms of orientation, depth, and gain to avoid LA foreshortening and to visualize the entire LA throughout the cardiac cycle. Five cardiac cycles of each plane were stored in cine loop format in order to subsequently select the images of better quality for off-line speckle-tracking analysis.

### Conventional echocardiography analysis

**Fig. 1:**
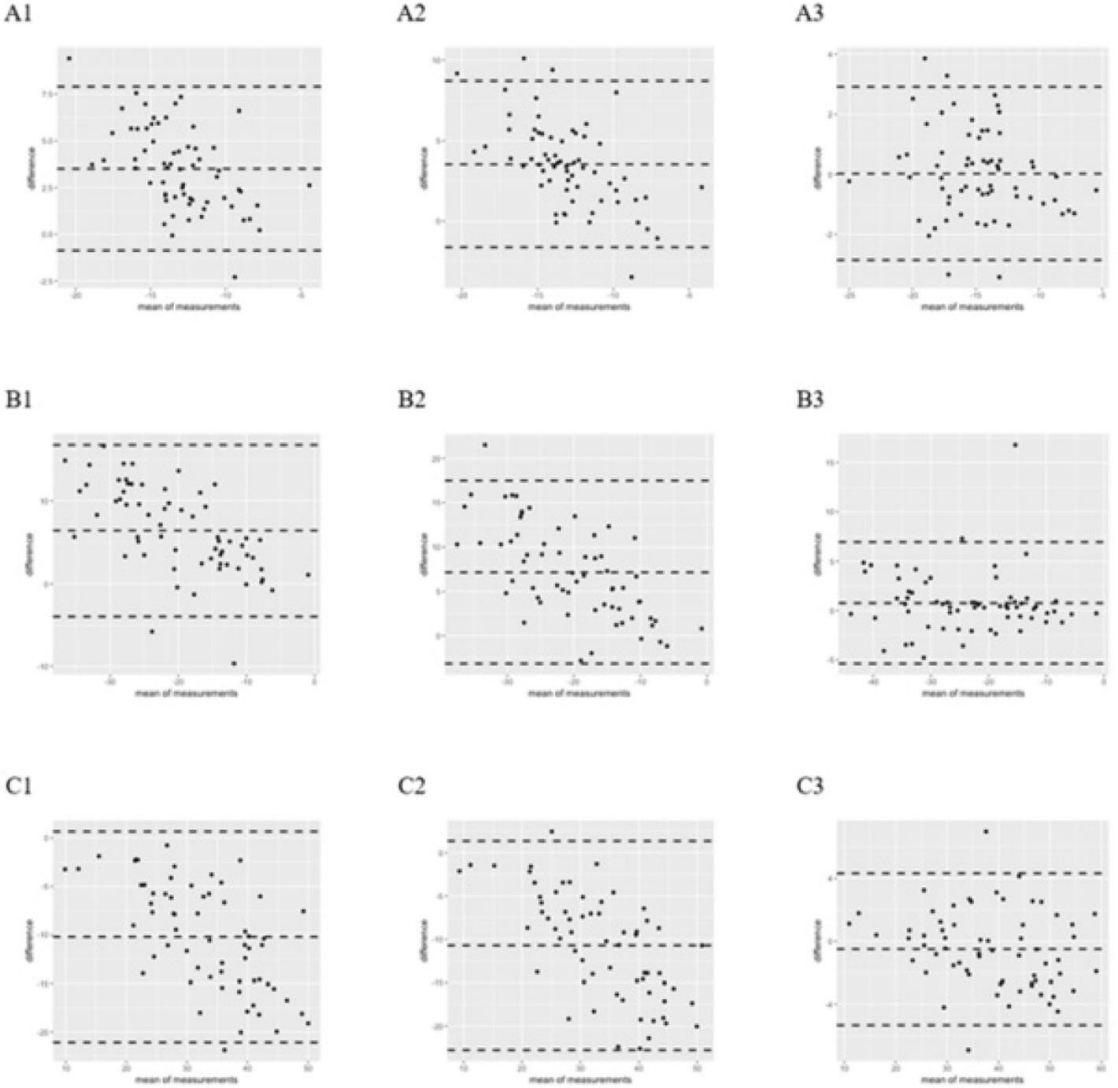
Bland-Altman scatter diagram of strain among VSS_LV_, VIS_LV_, and VIS_LA._ Compared with VIS, VSS had a larger bias and a wide 95% consistency range. Within VIS, the bias was small and the consistency range was relatively narrow. Sct: VSS_LV_ - VIS_LV_ (**A1**), VSS_LV_ - VIS_LA_ (**A2**), VIS_LV_ - VIS_LA_ (**A3**). Scd: VSS_LV_ - VIS_LV_ (**B1**), VSS_LV_ - VIS_LA_ (**B2**), VIS_LV_ - VIS_LA_ (**B3**). Sr: VSS_LV_ - VIS_LV_ (**C1**), VSS_LV_ - VIS_LA_ (**C2**), VIS_LV_ - VIS_LA_ (**C3**)

Offline analysis of images was performed using EchoPAC version 201 (GE Vingmed Ultrasound) (VSS_LV_) software, and Image Arena 2D Cardiac Performance Analysis version 4.6 (TomTec Imaging Systems, Unterschleissheim, Germany) (VIS_LA_ and VIS_LV_) software, yielding 3 strain analysis sets for each examination (Fig. 1).

From the parasternal long-axis view, LV end-diastolic diameter and LV end-systolic diameter were obtained at the level of the mitral valve tips by M-mode Doppler ultrasonography. Left ventricle ejection fraction (LVEF) was calculated automatically by the GE Vivid 9 system. From the apical 4- and 2-chamber positions, maximum LA volume (LAV_*max,*_; measured on the 2D frame just before mitral valve opening), LA pre-atrial contraction volume (LAV_*preA*_; measured on the frame just before the onset of atrial emptying), and LA minimum volume (LAV_*min,*_; measured on the frame at end-diastole with the smallest LA volume) were computed separately following American Society of Echocardiography guidelines, and using the biplane modified Simpson’s method of discs. The indices and formulas calculated from the volumes were as follows. Total LA stroke volume = LAV_*max*_ − LAV_*min*_. Active LA stroke volume = LAV_*preA*_ − LAV_*min*_. Passive LA stroke volume = LAV_*max*_ − LAV_*preA*_. Active LA emptying fraction = (active LA stroke volume / LAV_*preA*_) × 100%. Passive LA emptying fraction = (passive LA stroke volume / LAV_*max*_) × 100%. The LA expansion index = (total LA stroke volume / LAV_*min*_) × 100%. Pulsed wave Doppler at the apical position was used to obtain mitral inflow velocity between the tips of the mitral leaflets. E/e′ was calculated as early mitral inflow velocity (E) divided by the average of septal and lateral mitral annular peak early diastolic velocity (e′) obtained by pulsed wave tissue Doppler imaging (TDI).

### Speckle-tracking echocardiography analysis

Speckle-tracking analysis was performed by the software: VSS_LV_, VIS_LA_, and VIS_LV_. VSS_LV_ analyzed the image derived from raw data, while VIS_LV_ and VIS_LA_ analyzed the image from compressed data.

According to the consensus document of the EACVI/ASE/Industry Task Force recommendation [21], the LA was traced as followings: starting tracing the LA endocardial border at the endocardial border of the mitral annulus, up to the opposite mitral annulus side, while carefully excluding the pulmonary veins and LA appendage orifices. The apical two-chamber view was also analysed to obtain a biplane calculation of the LA strain.

The endocardial border was traced manually or was user defined. The user could review the tracking path and manually adjusted it after running. The adjustable numbers of images were 1/2 of the frame frequency with VIS_LA_ and VIS_LV_, and 1 frame with VSS_LV_. With VSS_LV_ and VIS_LV,_ the tracking location marker was placed at the end of QRS wave. With VIS_LA,_ the marker was placed at the beginning of the P wave. With VIS_LV_ and VIS_LA_, the amplitude of the ECG cannot be modulated.

A region of interest (ROI) was selected in the endocardial mode of VSS_LV_ and VIS_LV_, while it was defaulted to include the endocardium with VIS_LA_. VSS_LV_ uses full thickness ROI including endocardium and epicardium, the position and size of which can be adjusted. And the ROI tracing line of VIS is single, which can not be adjusted.

The software algorithms automatically performed speckle-tracking on a frame-to-frame basis.

Exclusion of the individual studies was done upon visual assessment, when abnormal curves were believed to be artefactual. Strain values were obtained from the apical 4- and apical 2-chamber views.

Intra-observer and inter-observer reproducibility of LA strain values were analyzed with repeated measurements by the same observer at 2 different time points, and by a second independent different observer. All observers were blinded to the results of the other software package and previous strain results when assessing reproducibility.

### Statistical analysis

Statistical analysis was performed using SPSS version 20 (IBM, Armonk, New York) and Empower (R) (www.empowerstats.com, X&Y Solutions, Inc., Boston MA) and R (http://www.R-project.org). All measurements were tested for distribution normality with the Kolmogorov–Smirnov test. Continuous variables were expressed as mean ± standard deviation (SD). Categorical variables were reported as percentages. Variability values were expressed by the coefficient of variation (CV), defined as CV = S/X × 100%, where S is the standard deviation and X is the mean value. Differences between groups were analyzed for statistical significance with the unpaired t-test or Mann-Whitney U test, as appropriate. Agreement between the 3 speckle-tracking methods was assessed by Bland–Altman analysis. The bias (mean difference) and the 95% limits of agreement (2 SDs around the mean difference) between the measurements derived from each system were calculated. The reliability for inter-software, intra-observer, and inter-observer measurements was evaluated by intra-class correlation coefficient (ICC) [22]. An ICC of ≥0.90 was considered excellent reliability, an ICC of ≥0.70 - < 0.90 was considered good reliability, an ICC of ≥0.50 - < 0.70 was considered moderate reliability, an ICC of ≥0.30 - < 0.50 was considered poor reliability, and an ICC of > 0.30 was considered very poor reliability. Pearson’s correlation coefficient and the point-biserial correlation coefficient were used to assess the correlation between strain value and baseline clinical characterizes. The Z-test (after transformation) was used to test the difference of correlation coefficient among the 3 software. All statistical tests are 2-sided, and a *p*-value < 0.05 is considered statistically significant.

## Results

All study subjects showed normal systolic function as determined by LVEF. However, the AF group had a significantly larger LA volume index (24.4 ± 5.9 vs. 31.7 ± 11.3, *p* = 0.002), LA volume (39.6 ± 10.8 vs. 55.7 ± 20.9 for LAV_max_, *p* < 0.001; 15.3 ± 6.1 vs. 25.8 ± 15.5, *p* < 0.001 for LAV_min_; 21.9 ± 7.9 vs. 37.3 ± 18.5 for LAV_preA_, *p* < 0.001), lower passive LA emptying fraction (%) (44.9 ± 10.2 vs. 34.3 ± 13.2, *p* < 0.001), lower LV emptying fraction (%) (61.7 ± 8.6 vs. 55.2 ± 12.8, *p* = 0.020), and lower LA expansion index (%) (176.0 ± 73.4 vs. 140.3 ± 65.8, *p* = 0.045) (Table 1).

**Table 1:** General characteristics of the study population. Clinical and echocardiographic features of the study population

Parameters	Normal group (*n* = 32)	AF group (*n* = 32)	Total (*n* = 64)	*P*
Age(y)	32.3 ± 10.3	55.9 ± 11.5	44.1 ± 16.1	< 0.001
Hight (cm)	162.2 ± 6.3	165.8 ± 7.2	164.0 ± 7.0	0.039
Weight (kg)	58.1 ± 9.6	68.7 ± 12.4	63.4 ± 12.2	< 0.001
Body weight index (kg/m^2^)	21.9 ± 2.4	24.9 ± 3.3	23.4 ± 3.2	< 0.001
Body surface area(m^2^)	1.6 ± 0.2	1.8 ± 0.2	1.7 ± 0.2	< 0.001
Systolic pressure (mmHg)	111.5 ± 7.9	132.3 ± 20.8	121.9 ± 18.8	< 0.001
Diastolic pressure (mmHg)	72.3 ± 6.2	75.3 ± 12.4	73.8 ± 9.8	0.224
2-chamber heart rate (bpm)	68.1 ± 9.4	66.0 ± 11.9	67.1 ± 10.7	0.445
4-chamber heart rate (bpm)	66.3 ± 9.8	67.9 ± 11.7	67.1 ± 10.7	0.549
LVDD (mm)	44.8 ± 3.9	45.8 ± 4.7	45.3 ± 4.3	0.330
LVSD (mm)	28.3 ± 3.6	28.8 ± 3.6	28.5 ± 3.6	0.581
E (cm/s)	0.9 ± 0.2	0.7 ± 0.2	0.8 ± 0.2	0.001
A (cm/s)	0.5 ± 0.1	0.7 ± 0.2	0.6 ± 0.2	0.006
E/A	1.8 ± 0.6	1.2 ± 0.5	1.5 ± 0.6	< 0.001
E/e’	7.8 ± 1.9	9.9 ± 3.8	8.8 ± 3.2	0.006
LVEF(%)	66.8 ± 4.6	66.4 ± 7.3	66.6 ± 6.1	0.792
LVMI(g/m^2^)	66.7 ± 12.0	91.4 ± 21.6	79.0 ± 21.4	< 0.001
LAD (mm)	30.3 ± 3.4	36.8 ± 5.3	33.5 ± 5.5	< 0.001
LAVI (ml/m^2^)	24.4 ± 5.9	31.7 ± 11.3	28.1 ± 9.7	0.002
LAV_max_ (ml)	39.6 ± 10.8	55.7 ± 20.9	47.6 ± 18.4	< 0.001
LAV_min_ (ml)	15.3 ± 6.1	25.8 ± 15.5	20.6 ± 12.8	< 0.001
LAV_preA_ (ml)	21.9 ± 7.9	37.3 ± 18.5	29.6 ± 16.1	< 0.001
Active LA emptying fraction (%)	30.1 ± 11.8	31.4 ± 14.4	30.8 ± 13.1	0.695
Passive LA emptying fraction (%)	44.9 ± 10.2	34.3 ± 13.2	39.6 ± 12.9	< 0.001
LV emptying fraction (%)	61.7 ± 8.6	55.2 ± 12.8	58.4 ± 11.3	0.020
LA expansion index(%)	176.0 ± 73.4	140.3 ± 65.8	158.1 ± 71.5	0.045
Stroke	0	1 (3.1)	1 (1.6)	1.000
Coronary heart disease	0	6 (18.8)	6 (9.4)	0.024
Diabetes	0	2 (6.3)	2 (3.1)	0.492
Hyperlipidemia	1 (3.1)	3 (9.4)	4 (6.3)	0.613
Hypertension				< 0.001
No	32 (100.0)	16 (50.0)	48 (75.0)	
Level 1	0	2 (6.3)	2 (3.1)	
Level 2	0	6 (18.8)	6 (9.4)	
Level 3	0	8 (25.0)	8 (12.5)	

*LVDD* left ventricular end diastolic diameter, *LVSD* left ventricular end systolic diameter, *LVEF* left ventricle ejection fraction, *LVMI* left ventricular mass index, *LAD* left atrial diameter, *LAVI* left atrial volume index, *LAV*_*max*_ maximum left atrial volume, *LAV*_*min*_ left atrial minimum volume, *LAV*_*preA*_ left atrial pre-atrial contraction volume

The mean heart rate showed no significant differences when strain was analyzed with 2-chamber view and 4-chamber view (67.1 ± 10.7 vs. 67.1 ± 10.7 beats per min [bpm], *p* = 1.00). As shown in Additional file 1: Tables S1–S3, the correlation coefficients between strain value and patient’s baseline clinical characteristics were calculated and compared among the software: VSS_LV_, VIS_LV_, and VIS_LA_. To assess the comparability between paired software (VSS_LV_-VIS_LV_, VSS_LV_-VIS_LA,_ and VIS_LV_-VIS_LA_), the overall ratio of non-significance was calculated for each specific software, and for each pair. For VSS_LV_, VIS_LV_, and VIS_LA_, the ratios were 92.86, 96.03, and 96.83%, respectively. For the pairs VSS_LV_-VIS_LV_, VSS_LV_-VIS_LA,_ and VIS_LV_-VIS_LA_, the ratios were 92.05, 93.65, and 100%, respectively.

### Strain analysis

Strain analysis was obtained in all 64 subjects. Representative examples of strain measured by the 3 systems are shown in Table 2. When using VSS_LV_, there were significant differences in intra-observer measurement of Sct and Sr, and inter-observer measurement of Sct (*p* < 0.05). Comparing VIS_LV_ and VIS_LA_, there were no significant differences in intra-observer or inter-observer measurements (*p* > 0.05).

**Table 2:** Left atrial strain measured by different observers (mean ± SD, *n* = 64)

	Sct(%)	Scd(%)	Sr(%)
VSS_LV_			
A1	−11.52 ± 2.69	−16.96 ± 7.28	28.49 ± 7.82
A2	− 11.14 ± 2.37^*^	−16.71 ± 7.10	27.85 ± 7.65^*^
B	− 11.12 ± 2.60^#^	−16.63 ± 7.24	27.75 ± 7.77
Mean	− 11.26 ± 2.46	− 16.77 ± 7.06	28.03 ± 7.58
*P*	0.004	0.244	0.007
VIS_LV_			
A1	−14.54 ± 3.72	−23.35 ± 10.63	37.89 ± 11.89
A2	−14.64 ± 3.67	−22.81 ± 9.95	37.46 ± 10.83
B	−15.14 ± 3.99	−24.21 ± 10.58	39.35 ± 11.58
Mean	− 14.77 ± 3.59	− 23.17 ± 10.33	38.23 ± 10.99
*P*	0.610	0.109	0.261
VIS_LA_			
A1	− 14.90 ± 4.13	− 23.72 ± 10.83	38.62 ± 12.19
A2	−14.72 ± 4.03	− 23.73 ± 10.57	38.45 ± 11.69
B	−14.77 ± 3.83	− 24.36 ± 10.95	39.13 ± 11.77
Mean	−14.80 ± 3.88	− 23.94 ± 10.49	38.73 ± 11.56
*P*	0.264	0.967	0.607

When using VSS_LV_, there were significant differences in intra-observer measurement of Sct and Sr, and inter-observer measurement of Sct (*p* < 0.05). Comparing VIS_LV_ and VIS_LA_, there were no significant differences in intra-observer or inter-observer measurements (*p* > 0.05)

A1: First measurement made by observer A

A2: Second measurement made by observer A 1 month later

B: First measurement made by observer B

^#^Inter-observer comparison of VSS_LV_, VIS_LV_, and VIS_LA_ (*p* < 0.05)

^*^Intra-observer comparison of VSS_LV_, VIS_LV_, and VIS_LA_ (*p* < 0.05)

Strain measurements between VSS_LV_ and VIS (VIS_LV_ and VIS_LA_) demonstrated considerable variability in Sct, Scd and Sr as assessed by their CoV, as shown in Table 3, the CoV for measurements between VSS_LV_ and VIS (VIS_LA_ and VIS_LV_) ranged from 0.04 to 31.16% (Sct), − 1.94 to 38.21%(Scd) and − 1.29 to 36.40%(Sr).

**Table 3:** The CoV for the values measured by VSS and VIS (*n* = 64)

		Sct	Scd	Sr
VSS_LV_	VIS_LV_	31.16%	38.21%	36.40%
VSS_LV_	VIS_LA_	31.37%	42.76%	38.19%
VIS_LV_	VIS_LA_	0.04%	−1.94%	−1.29%

**Fig. 2:**
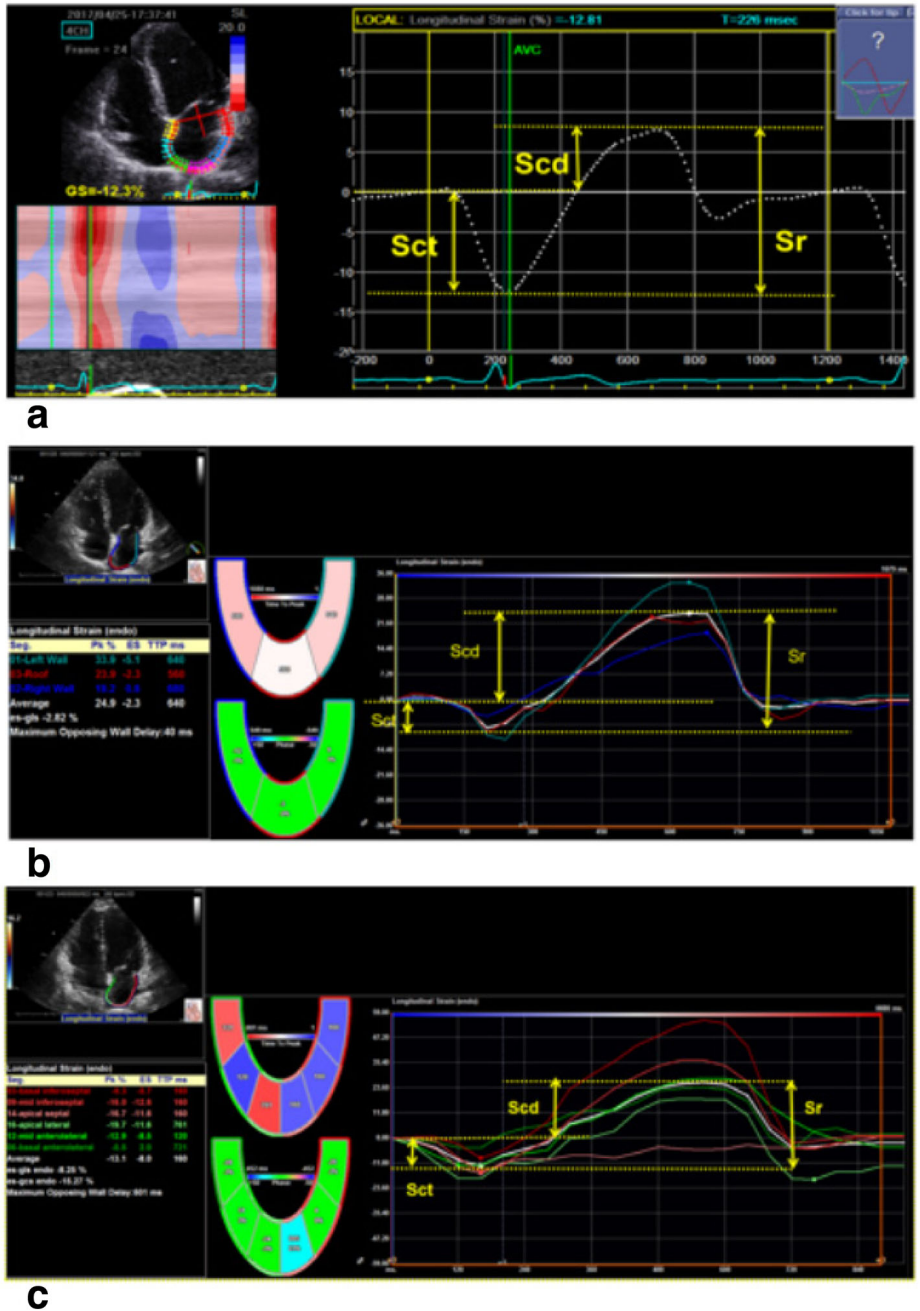
Schematic diagram of left atrium global longitudinal strain index of measured by VSS_LV_, VIS_LV_ and VIS_LA_. Apical four-chamber view was obtained using conventional 2D echocardiography. The left atrial strain (**a**) measured by VSS_LV_; The left atrial strain (**b**) measured by VIS_LA_;the left atrial strain (**c**) measured by VIS_LV_. The white dashed line (**a**) and white lines (**b** and **c**) represent the average strain. r, reservoir phase; cd, conduit phase; ct, contraction phase. The respective strains are Sr, calculated as difference between onset of filling and end-diastole (positive value); Scd, calculated as difference between onset of atrial contraction and onset of filling (negative value); Sct, calculated as difference between end-diastole and onset of atrial filling (negative value)

The comparability of strain measurements showed good agreement within VIS (VIS_LA_ and VIS_LV_), with a smaller mean differences, which were 0.02 (Sct), 0.76 (Scd), and − 0.50 (Sr), and a narrower limits of agreement ranging from − 5.42 to 7.05. Between VSS_LV_ and VIS_LV,_ mean differences were 3.51 (Sct), 6.41 (Scd), and − 10.20(Sr), with limits of agreement ranging from 16.99 to − 21.30. Between VSS_LV_ and VIS_LA_, the mean differences were 3.53 (Sct), 7.17 (Scd), and − 10.70 (Sr), with limits of agreement ranging from 17.69 to − 23.03. Figure 2 shows the Bland-Altman Scatter plots.

In Table 4, the intra-class correlation coefficients revealed excellent reliability for VIS, with the correlation coefficients generally > 0.9.

**Table 4:** Intra-class correlation coefficients of the different software (*n* = 64)

		Sct	Scd	Sr
VSS_LV_	VIS_LV_	0.31	0.61	0.42
VSS_LV_	VIS_LA_	0.29	0.58	0.39
VIS_LV_	VIS_LA_	0.92	0.95	0.98

All of the ICCs reached statistical significance (all, *p* < 0.01). Based on the correlation coefficient, there was good reliability between VIS_LV_ and VIS_LA_

Overall, there were fewer differences between strain values from VIS compared with strain values from VSS derived from same images.

### Inter-observer and intra-observer variability and correlation in the determination of LA strain

As shown in Table 5, the inter-observer and intra-observer variability (mean and range) of the measurements derived from VSS_LV_ were 2.35% (1.52–3.30%) and 2.67% (1.95–3.49%), respectively. The differences of inter-observer measurement of Sct and Sr were statistically significant (*p* < 0.05), and the intra-observer difference of measurement of Sct was significant (*p* < 0.05). The inter-observer and intra-observer variability (mean and range) of VIS_LV_ were 1.39% (0.71–2.31%) and 3.87% (3.67–4.10%), respectively. Both intra-observer and inter-observer variability were not significantly different (*p* = 0.01).

**Table 5:** Intra-observer and inter-observer CoVs for strain value measured by the same software (*n* = 64)

	Intra-observer			Inter-observer		
	Sct	Scd	Sr	Sct	Scd	Sr
VSS_LV_	3.30%	1.52%	2.24%	3.49%	1.95%	2.57%
VIS_LV_	0.71%	2.31%	1.15%	4.10%	3.67%	3.83%
VIS_LA_	1.18%	0.05%	0.43%	0.85%	2.68%	1.32%

Intra-observer and inter-observer CoVs for differences of Sct and Sr measured by VSS_LV_ were significant (both, *p* < 0.05)

Intra-observer and inter-observer CoVs for differences of Sct measured by VIS_LV_ had no significant differences (*p* > 0.05)

Intra-observer and inter-observer CoVs for differences of strain measured by VIS_LA_, were of no significant differences (*p* > 0.05)

For VIS_LA_, the inter-observer and intra-observer variability (mean and range) were 0.55% (0.05–1.18%) and 1.62% (0.85–2.68%), respectively, and there were no significant differences (*p* > 0.05).

The results of the analysis of bias and limits of agreement for the same software are shown in Table 6. The bias for value measurement by VIS_LA_ was smaller than that by VSS_LV_ and VIS_LV_.

**Table 6:** Bias and limits of agreement for the same software (*n* = 64)

	Intra-observer			Inter-observer		
VSS_LV_	−0.38 (−2.44, 1.68)	− 0.26 (−3.76, 3.24)	0.64 (− 3.05, 4.33)	− 0.40 (− 3.31, 2.50)	−0.33 (−5.35, 4.68)	0.73 (−5.37, 6.84)
VIS_LV_	0.10 (− 3.11, 3.31)	− 0.54 (− 5.86, 4.78)	0.44 (− 5.73, 6.60)	0.60 (−4.45, 5.65)	0.86 (−11.43, 13.14)	−1.45 (− 14.81, 11.90)
VIS_LA_	− 0.18 (− 2.68, 2.32)	0.01 (− 4.12, 4.15)	0.17 (− 4.95, 5.28)	−0.13 (− 3.98, 3.73)	0.64 (−9.84, 11.11)	−0.51 (− 11.66, 10.64)

The bias for value measurement by VIS_LA_ was smaller than that by VSS_LV_ and VIS_LV_

The inter-observer and intra-observer evaluation results of the same software for intra-class correlation coefficient are shown in Table 7. The inter-observer ICC values were VIS_LA_ > VSS_LV_ = VIS_LV_; for intra-observer ICC, VSS_LV_ > VIS_LA_ > VIS_LV_.

**Table 7:** Intra-observer and inter-observer intra-group correlation coefficients for the same software (*n* = 64)

	Intra-observer				Inter-observer			
	Sct	Scd	Sr	Mean (range)	Sct	Scd	Sr	Mean (range)
VSS_LV_	0.91	0.97	0.97	0.95 (0.91–0.97)	0.84	0.94	0.92	0.90 (0.84–0.94)
VIS_LV_	0.91	0.97	0.96	0.95 (0.91–0.97)	0.78	0.83	0.83	0.81 (0.78–0.83)
VIS_LA_	0.95	0.98	0.98	0.97 (0.95–0.98)	0.88	0.88	0.89	0.88 (0.88–0.89)

All the ICCs reached significant (*P* < 0.01)

## Discussion

In this study, we sought to investigate the reproducibility of various LA strain analyses obtained using VSS (VSS_LV_) and VIS (VIS_LV_ and VIS_LA_), and evaluate the agreement between the methods. The main results of this study are that (1) when comparing VSS (VSS_LV_) and VIS (VIS_LV_ and VIS_LA_), the absolute values of the CoV for strain measured by VSS were larger than those measured by VIS. In addition, the ICC between VIS and VSS indicated that the measurement reliability was weak (generally < 0.6). However, when comparing strain measured between VIS_LA_ and VIS_LV_, the bias and CV were smaller, and reliability was good. Therefor measuring LA strain across the different vendors is not recommended. (2) The reproducibility of inter-observer and intra-observer measurement within the same software was good. The CoV were minor and < 10% [18], and Bland-Altman analysis suggested that the bias of 3 software was small and the ranges of consistency were stable. VSS have no obvious advantages compared with VIS, and measurements derived from VIS_LA_ had the smallest bias. (3) Strain values were correlated with patient baseline clinical characteristics. The measurements obtained with the same software were more consistent than those obtained with different software, which might influence the analysis of the results clinically. These findings are important in view of future clinical applications of 2D-STE, particularly for patient diagnosis and follow-up in centers where there are a diversity of cardiac ultrasound systems.

In a study of similar design focusing on LV and RV strain [23], the authors found there was good reproducibility for global longitudinal strain, but only moderate reproducibility for circumferential strain and poor reproducibility for radial strain when comparing LV strain, and good reproducibility across different ultrasound platforms and software packages when comparing the RV strain [24]. The main contractile direction of the left atrium is longitudinal [25], and it is reasonable to posit that Sct had a smaller bias and CoV across different ultrasound platforms and software packages than Sr and Scd. The strain values measured were generally correlated with baseline clinical characteristics. However, when measuring Scd and Sr with different software, differences of the correlation coefficients were prominent. And even with improved observer reliability, the intra-observer differences between Scd and Sr measured by VIS_LV_ and VIS_LA_ was still significant, while there was no significant intra-observer difference in the repeated measurements by the same software. A consensus has been reached that the relative variation in strain measurements among different software should not exceed 10% [18]. We believe 10% is a proper reference, and found the variations between VIS and VSS ranged from 31.16 to 42.76%, while that compared within VIS did not exceed 10%. Therefore, we suggest different software are not interchangeable when analyzing 2D strain data to assess LA strain from the same subjects.

Compared with measurements derived from VIS, those from VSS had large bias and a wide 95% consistency range, while within VIS the bias was small and the consistency range was relatively narrow. We believe the differences of CoV and bias between VIS and VSS are due to dissimilarity in ROI [26, 27]. During tracing of the left atrium, VSS tracks speckles from the mid-myocardium, while VIS determines strain from the endocardium. Therefore, a full-thickness ROI will be placed by VSS. With VIS, the line placed is slightly within the endocardial wall. Variation in exact placement of the ROI will cause variation in longitudinal strain values, as longitudinal strain decreases from the endocardium to the epicardium [28, 29]. It is also important to know that the different software use different algorithms to calculate deformation and express the results. However, the algorithms are not publically disclosed.

Different from the results of the research conducted by Takigiku et al. [30], that showed intra- and inter-observer ICCs were always better than inter-vendor agreement, our study showed that VSS had the best inter-observer ICCs, while VIS_LA_ had the best intra-observer ICCs. We supposed the repeatability of zero strain chosen at the beginning of the p-wave could cause the differences [27, 31]. GE has the access to the raw data, while TomTec is disallowed from the manufacturers’ raw data. Therefor VIS analyses the strain by the data which were compressed, which could decrease the repeatability of zero strain selection and increase the error of strain output. However, with improved operator experience, it is possible to improve the repeatability of the zero strain setting to a certain extent by correcting the starting point of LA contraction with the 2D image [27]. Furthermore, with VIS_LA_ the tracking location marker was placed at the starting frame of the p-wave before the LA contraction began, while with VSS the marker was placed at the end of QRS where the LA contraction has started. Therefore, the left atrium will be more dilated in VIS_LA_, which makes tracking more accurate by avoiding the entrance of the pulmonary veins and LA appendix [32]. Even though VSS_LV_ has the access to the raw data (before Digital Imaging and Communications in Medicine [DICOM] formatting), while VIS_LV_ and VIS_LA_ are disallowed from the manufacturers’ raw data and only gets access to the DICOM, VSS had no obvious advantages compared with VIS, and measurements derived from VIS_LA_ had the smallest bias.

## Conclusion

Measurement of LA strain is an evolving echocardiographic technique for the assessment of LA function, and has been studied in a variety of clinical settings [33]. The recent European Association of Cardiovascular Imaging and the American Society of Echocardiography 2018 task force highlighted that differences between vendor software for strain assessment remain a very important barrier to widespread use and applicability of LA strain. To our knowledge, there are still no data showing a variability between different ultrasound software packages regarding LA analyses. And this is the first study focusing on discussing the reproducibility of various LA strain analyses obtained by different software packages. The findings of our study reveals when comparing VSS (VSS_LV_) and VIS (VIS_LV_ and VIS_LA_), the absolute values of the CoV for strain measured by VSS were larger than those measured by VIS, therefor we suggest that the same 2D-STE software should be used to analysis the left atrial strain during patients’ following up. Since we find measurements derived from VIS_LA_ had the smallest bias, we think the dedicated 2D-STE software should be used to analyze LA strain.

### Limitations

The study was not designed to assess the accuracy of LA strain measurements as there was no comparison to gold standard. This study was focused on determining the reproducibility of LA strain measurements among different vendors.

We used small numbers of subjects to assess consistency among 3 vendors. However there are no data showing a variability between different ultrasound software packages regarding LA analyses, and the use of a larger number of subjects may help clarify this issue.

Although we included patients with AF, they were in sinus rhythm during analyzing and further studies are needed to assess use of LA strain in patients who are not in sinus rhythm.

We excluded the patients with poor image quality. However it is important for STE analyzing, prospective image acquisition with a focus on LA optimisation would benefit further LA strain study.

## Additional file

Additional file 1: **Table S1.** Correlation coefficients and their paired-comparisons among software. **Table S2**. Correlation coefficients and their paired-comparisons among software for Scd. **Table S3**. Correlation coefficients and their paired-comparisons among software for Sr (DOCX 21 kb)

## Abbreviations

2D-STE: Two-dimensional speckle-tracking echocardiography
CoV: Coefficient of variation
EF: Ejection fraction
ICCs: Intra-class correlation coefficients
LA: Left atrial
LV: Left ventricle
ROI: Region of interest
Scd: Strain during conduit phase
Sct: Strain during the contraction phase
Sr: Strain during reservoir phase
VIS: Vendor-independent software packages
VSS: Vendor-specific software
