# Supplementary material for: Left atrial strain reproducibility using vendor-dependent and vendor-independent software
Source: Cardiovasc Ultrasound. 2019 May 15;17:9. doi: 10.1186/s12947-019-0158-y (PMC6521472; doi:10.1186/s12947-019-0158-y)
Supplement: Supplementary file 1 — Table S1. Correlation coefficients and their paired-comparisons among software. Table S2. Correlation coefficients and their paired-comparisons among software for Scd. Table S3. Correlation coefficients and their paired-comparisons among software for Sr (DOCX 21 kb) [file 12947_2019_158_MOESM1_ESM.docx]

Supplementary Table 1 Correlation coefficients and their paired-comparisons among software for Sct

|  | Sct | | |  | Paired-comparisons (Z-score) | | |
| --- | --- | --- | --- | --- | --- | --- | --- |
| Parameters | VSS_LV_ | VIS_LV_ | VIS_LA_ |  | VSS_LV_ - VIS_LV_ | VSS_LV_ - VIS_LA_ | VIS_LV_ - VIS_LA_ |
| Systolic pressure(mmHg) | -0.078 | -0.004 | 0.011 |  | 0.58 | 0.70 | 0.12 |
| Diastolic pressure(mmHg) | -0.218 | -0.144 | -0.146 |  | 0.61 | 0.59 | -0.01 |
| LVDD(mm) | 0.116 | 0.184 | 0.132 |  | 0.53 | 0.12 | -0.42 |
| LVSD(mm) | 0.036 | 0.169 | 0.114 |  | 1.03 | 0.61 | -0.44 |
| E(cm/s) | 0.060 | -0.053 | -0.040 |  | -0.88 | -0.78 | 0.10 |
| A(cm/s) | -0.081 | -0.118 | -0.097 |  | -0.28 | -0.12 | 0.16 |
| E/A | 0.197 | 0.206 | 0.185 |  | 0.05 | -0.11 | -0.18 |
| E/e’ | -0.057 | -0.056 | -0.032 |  | 0.01 | 0.20 | 0.19 |
| LVEF(%) | 0.087 | -0.075 | -0.058 |  | -1.27 | -1.14 | 0.14 |
| LVMI(g/m^2^) | 0.057 | 0.139 | 0.104 |  | 0.64 | 0.36 | -0.29 |
| LAD(mm) | 0.181 | 0.265* | 0.232 |  | 0.64 | 0.38 | -0.31 |
| LAV_max_(ml) | 0.300* | 0.297* | 0.248* |  | -0.10 | -0.48 | -0.45 |
| LAV_min_(ml) | 0.480** | 0.481** | 0.440** |  | -0.33 | -0.64 | -0.66 |
| LAV_preA_ (ml) | 0.365** | 0.373** | 0.339** |  | -0.07 | -0.34 | -0.42 |
| Active LA emptying fraction (%) | -0.331** | -0.338** | -0.269* |  | 0.04 | 0.59 | 0.65 |
| Passive LA emptying fraction (%) | -0.231 | -0.301* | -0.291* |  | -0.51 | -0.44 | 0.15 |
| LV emptying fraction (%) | -0.459** | -0.497** | -0.447** |  | -0.01 | 0.38 | 0.77 |
| LA expansion index(%) | -0.361** | -0.425** | -0.369** |  | -0.37 | 0.07 | 0.66 |
| LAVI(ml/m^2^) | 0.330** | 0.301* | 0.243 |  | -0.33 | -0.78 | -0.53 |
| Cardiac risk factors | 0.084 | 0.263* | 0.301* |  | 1.40 | 1.70 | 0.25 |

LVDD: left ventricular end diastolic diameter; LVSD: left ventricular end systolic diameter; LVEF: left ventricle ejection fraction; LVMI: left ventricular mass index; LAD: left atrial diameter; LAVI: left atrial volume index; LAVmax: maximum left atrial volume; LAVmin: left atrial minimum volume; LAVpreA: left atrial pre-atrial contraction volume

* *p* < 0.05; ** *p* < 0.01.

Supplementary Table 2 Correlation coefficients and their paired-comparisons among software for Scd

|  | Scd | | |  | Paired-comparisons (Z-score) | | |
| --- | --- | --- | --- | --- | --- | --- | --- |
| Parameters | VSS_LV_ | VIS_LV_ | VIS_LA_ |  | VSS_LV_ - VIS_LV_ | VSS_LV_ - VIS_LA_ | VIS_LV_ - VIS_LA_ |
| Systolic pressure(mmHg) | 0.558** | 0.505** | 0.507** |  | -0.98 | -0.97 | -0.38 |
| Diastolic pressure(mmHg) | 0.305* | 0.189 | 0.184 |  | -0.99 | -1.03 | -0.06 |
| LVDD(mm) | 0.128 | 0.116 | 0.134 |  | -0.10 | 0.04 | 0.14 |
| LVSD(mm) | 0.031 | 0.067 | 0.087 |  | 0.28 | 0.43 | 0.16 |
| E(cm/s) | -0.582** | -0.573** | -0.559** |  | 0.71 | 0.82 | 0.73 |
| A(cm/s) | 0.331** | 0.321** | 0.322** |  | -0.18 | -0.18 | -0.09 |
| E/A | -0.594** | -0.593** | -0.591** |  | 0.71 | 0.73 | 0.71 |
| E/e’ | 0.222 | 0.273* | 0.304* |  | 0.37 | 0.61 | 0.19 |
| LVEF(%) | -0.027 | -0.078 | -0.081 |  | -0.39 | -0.42 | -0.03 |
| LVMI(g/m^2^) | 0.577** | 0.566** | 0.590** |  | -0.72 | -0.53 | -0.41 |
| LAD(mm) | 0.717** | 0.618** | 0.644** |  | -2.22* | -2.02* | -0.61 |
| LAV_max_(ml) | 0.603** | 0.526** | 0.519** |  | -1.34 | -1.39 | -0.51 |
| LAV_min_(ml) | 0.651** | 0.564** | 0.587** |  | -1.66 | -1.48 | -0.41 |
| LAV_preA_ (ml) | 0.704** | 0.618** | 0.642** |  | -2.00* | -1.82 | -0.63 |
| Active LA emptying fraction (%) | -0.078 | 0.005 | 0.000 |  | 0.65 | 0.61 | -0.04 |
| Passive LA emptying fraction (%) | -0.663** | -0.574** | -0.628** |  | 1.75 | 1.32 | 0.20 |
| LV emptying fraction (%) | -0.588** | -0.469** | -0.511** |  | 1.61 | 1.28 | -0.02 |
| LA expansion index(%) | -0.542** | -0.397** | -0.466** |  | 1.65 | 1.11 | -0.36 |
| LAVI(ml/m^2^) | 0.537** | 0.486** | 0.479** |  | -0.89 | -0.95 | -0.40 |
| Cardiac risk factors | 0.684** | 0.735** | 0.743** |  | -0.79 | -0.72 | -1.53 |

LVDD: left ventricular end diastolic diameter; LVSD: left ventricular end systolic diameter; LVEF: left ventricle ejection fraction; LVMI: left ventricular mass index; LAD: left atrial diameter; LAVI: left atrial volume index; LAVmax: maximum left atrial volume; LAVmin: left atrial minimum volume; LAVpreA: left atrial pre-atrial contraction volume

* *p* < 0.05; ** *p* < 0.01.

Supplementary Table 3 Correlation coefficients and their paired-comparisons among software for Sr

|  | Sr | | |  | Paired-comparisons (Z-score) | | |
| --- | --- | --- | --- | --- | --- | --- | --- |
| Parameters | VSS_LV_ | VIS_LV_ | VIS_LA_ |  | VSS_LV_ - VIS_LV_ | VSS_LV_ - VIS_LA_ | VIS_LV_ - VIS_LA_ |
| Systolic pressure(mmHg) | -0.495** | -0.455** | -0.463** |  | 0.68 | 0.61 | 0.22 |
| Diastolic pressure(mmHg) | -0.213 | -0.167 | -0.118 |  | 0.38 | 0.77 | 0.40 |
| LVDD(mm) | -0.157 | -0.144 | -0.166 |  | 0.11 | -0.06 | -0.16 |
| LVSD(mm) | -0.041 | -0.115 | -0.117 |  | -0.58 | -0.60 | -0.02 |
| E(cm/s) | 0.522** | 0.536** | 0.521** |  | -0.33 | -0.45 | -0.61 |
| A(cm/s) | -0.282* | -0.262* | -0.259* |  | 0.22 | 0.24 | 0.07 |
| E/A | 0.489** | 0.481** | 0.474** |  | -0.43 | -0.48 | -0.39 |
| E/e’ | -0.188 | -0.244 | -0.266* |  | -0.42 | -0.59 | -0.13 |
| LVEF(%) | -0.003 | 0.080 | 0.093 |  | 0.64 | 0.75 | 0.10 |
| LVMI(g/m^2^) | -0.556** | -0.567** | -0.570** |  | 0.47 | 0.44 | 0.57 |
| LAD(mm) | -0.727** | -0.667** | -0.662** |  | 1.99* | 2.03* | 1.12 |
| LAV_max_(ml) | -0.658** | -0.568** | -0.554** |  | 1.73 | 1.84 | 0.71 |
| LAV_min_(ml) | -0.761** | -0.682** | -0.681** |  | 2.48* | 2.49* | 1.19 |
| LAV_preA_ (ml) | -0.773** | -0.706** | -0.696** |  | 2.52* | 2.60** | 1.43 |
| Active LA emptying fraction (%) | 0.180 | 0.076 | 0.091 |  | -0.83 | -0.71 | 0.11 |
| Passive LA emptying fraction (%) | 0.692** | 0.679** | 0.668** |  | -1.35 | -1.44 | -1.25 |
| LV emptying fraction (%) | 0.696** | 0.616** | 0.613** |  | -1.91 | -1.93 | -0.82 |
| LA expansion index(%) | 0.622** | 0.528** | 0.546** |  | -1.56 | -1.42 | -0.33 |
| LAVI(ml/m^2^) | -0.607** | -0.532** | -0.516** |  | 1.35 | 1.47 | 0.60 |
| Cardiac risk factors | -0.664** | -0.743** | -0.775** |  | 0.44 | 0.19 | 1.42 |

LVDD: left ventricular end diastolic diameter; LVSD: left ventricular end systolic diameter; LVEF: left ventricle ejection fraction; LVMI: left ventricular mass index; LAD: left atrial diameter; LAVI: left atrial volume index; LAVmax: maximum left atrial volume; LAVmin: left atrial minimum volume; LAVpreA: left atrial pre-atrial contraction volume

* *p* < 0.05; ** *p* < 0.01.
